# Leveraging a Strategic Public–Private Partnership to Launch an Airport-Based Pathogen Monitoring Program to Detect Emerging Health Threats

**DOI:** 10.3201/eid3113.241407

**Published:** 2025-05

**Authors:** Cindy R. Friedman, Robert C. Morfino, Ezra T. Ernst

**Affiliations:** Centers for Disease Control and Prevention, Atlanta, Georgia, USA (C.R. Friedman); Gingko Bioworks, Boston, Massachusetts, USA (R.C. Morfino); Xwell, New York, New York, USA (E.T. Ernst)

**Keywords:** COVID-19, public–private partnerships, innovation, traveler-based genomic surveillance, airplane wastewater surveillance, pandemic preparedness, early detection, respiratory infections, viruses, SARS-CoV-2, variants, travelers, biosecurity, outbreak identification, whole genome sequencing, public health monitoring

## Abstract

Airport-based pathogen monitoring is a critical tool that can contribute to early detection and characterization of existing and new pathogen threats. A novel public–private partnership between an airport spa group, a biotech company, and the Centers for Disease Control and Prevention was instrumental in establishing a multimodal pathogen genomic surveillance program at US international airports. That public–private partnership addressed critical challenges that neither party could overcome independently, resulting in the development and deployment of a scalable, flexible early warning system for pathogen detection and public health monitoring.

The COVID-19 pandemic demonstrated the need for large-scale public health response mechanisms including surveillance, laboratory testing, and genomic sequencing (*1*). Public–private partnerships can be used to deploy those measures rapidly, especially during public health emergencies. During the pandemic, the Centers for Disease Control and Prevention (CDC) partnered with an airport spa company (XpresCheck, https://xprescheck.com) and a biotech firm (Ginkgo Biosecurity, https://biosecurity.ginkgo.bio) to develop an innovative traveler-based genomic surveillance system for early detection of new SARS-CoV-2 variants. The program expanded from an initial proof-of-concept pilot launched during September 2021 to a dynamic multiairport monitoring system during a few days in November 2021, just as the Omicron variant was identified (*2*).  International air travelers move rapidly across the globe, bringing the potential to spread communicable diseases, thereby making traveler-based public health surveillance a critical tool to gain early knowledge about the emergence and spread of existing and new pathogens. During the 20 years before the COVID-19 pandemic, global traveler-based public health surveillance programs were driven by more traditional collaborations with academic institutions and travel medicine clinics to conduct sentinel surveillance among travelers (*3*). Those programs provided insights and guidance for traveling populations and travel medicine clinicians (*3*). However, those programs relied on symptomatic travelers seeking medical care and clinicians ordering appropriate laboratory tests, which, if positive, might undergo whole-genome sequencing and be reported to public health. That screening process takes time and, early in the COVID-19 pandemic, its effectiveness was limited because nearly half of patients with SARS-CoV-2 infections were asymptomatic (*4*). As the pandemic evolved, many symptomatic persons might not have sought healthcare or might have used antigen-based self-tests that would not yield a sample for genomic sequencing; therefore, new variants emerging in one part of the world could go undetected while spreading globally (*5*). 

In early 2021, US public health authorities sought to quickly sequence samples from incoming international travelers at US airports—not for case identification or contact tracing, but to gain an early snapshot of new SARS-CoV-2 variants entering the country. Rapid detection of those variants, which had previously been challenging, was crucial for timely analysis and to help adjust mitigation strategies.

The preferences for the proposed genomic surveillance program included infrastructure for recruiting and sampling travelers in airports, voluntary traveler participation, and a rapid turnaround time for reporting results, including genomic sequences. The Centers for Disease Control and Prevention (CDC) learned about an airport-based company that pivoted from offering spa services, such as manicures and massages, to providing rapid COVID-19 testing for outbound US travelers needing to meet international entry requirements. A biotechnology company joined the initiative to contribute approaches for genomic sequencing. The resulting public–private partnership, the Traveler-based Genomic Surveillance Program, provided flexibility to adjust methods quickly—a key advantage of the collaboration. The partnership addressed a critical gap that none of the parties could fill independently: developing and deploying a scalable early warning system for public health genomic surveillance.

Public health agencies often lack the capacity to manage operational complexity at the scale that industry partners bring to the table. The collaboration we describe established a traveler-based genomic surveillance system that could adapt to the evolving pandemic, respond swiftly to emerging threats, and serve as a novel tool for outbreak detection and pandemic preparedness. For example, collecting samples from volunteer travelers required access to specific areas of the airport, security clearance for staff, developing a customized process for recruiting consenting travelers, and collecting and registering samples without interfering with airport operations, all while seamlessly integrating into the travelers’ journeys. 

When the program expanded to include environmental testing, collecting wastewater samples from aircraft necessitated creating a collection device and sampling process (*6*). That creation involved several design and testing cycles and negotiations with multiple stakeholders, including ground handlers, airport authorities, airlines, operations teams, and local public health agencies. The dynamic pandemic landscape required rapid operational scaling, including quick staff recruitment and increased testing capacity within hours in response to catalysts, such as rising case numbers in certain global regions, countries with limited sequencing capability, or newly identified variants (*7*). In addition, the program needed the ability to revert to baseline operations when the acute event concluded. In all scenarios, the private sector’s ability to rapidly develop a prototype, pilot test it, and execute new solutions at scale was crucial in enabling CDC to achieve its vision and objectives in this arena.

Over the next 3 years, this public–private partnership enabled expansion of traveler-based genomic surveillance that had tested >600,000 travelers and >1,200 wastewater samples across 10 airports by August 2024 (Table). During the expansion, fast turnaround time from sample collection to reporting was critical, so the partners built a process that provided reporting of PCR results within 24–48 hours and whole-genome sequencing within 10 days of collection. The Traveler-based Genomic Surveillance Program evolved into a multimodal platform that included nasal, aircraft wastewater, and air sampling and a comprehensive approach for multipathogen detection (*11*). The private sector played a crucial role in the evolution of the program through scaled technology deployment, rapid iterations in response to changing conditions, and extending reach into areas typically beyond the traditional scope of public health.

**Table:** Leveraging public–private partnerships to expand CDC’s Traveler-based Genomic Surveillance airport-based pathogen monitoring program, September 2021­–August 2024*

Milestones	2021		2022	2023	Jan–Aug 2024
	Sep 29–Nov 27	Nov 28–Dec 31			
Launch	Launched 6-week pilot, demonstrating operational feasibility and detection and genomic sequencing of SARS-CoV-2 in samples from travelers	Expanded pilot for Omicron surge; identified Omicron subvariants BA.2 and BA.3 six weeks before those variants were reported globally (*2*)	Launched airplane wastewater pilot at JFK (*5*); demonstrated retroactively that US predeparture test requirement during COVID-19 pandemic reduced postarrival positivity by 50% (*8*); enhanced surveillance for 2022 FIFA World Cup (*9*)	Expanded coverage of flights from China and surrounding hubs during China’s removal of its “zero-COVID” policy and subsequent surge of cases; detected first BA.2.86 in a traveler from Japan (*10*); detected FLiRT† mutations in wastewater samples 3 weeks before reported globally	Launched transatlantic airplane wastewater pilot in collaboration with United Kingdom Health Security Agency; enhanced surveillance during Hajj and 2024 Summer Olympics
Airports involved	EWR, JFK T4, SFO	ATL, EWR, JFK T4, SFO	ATL, EWR, IAD, JFK T4, SFO	ATL, BOS, EWR, IAD, JFK T4, JFK T8, LAX, SEA, SFO	BOS, EWR, IAD, JFK T4, JFK T8, LAX, MIA, SEA, SFO
Modalities	Nasal sampling in airport; at-home saliva sampling with questionnaire	Nasal sampling in airport; at-home saliva sampling with questionnaire	Nasal sampling in airport and traveler questionnaire; discontinued at-home saliva sampling; airplane wastewater sampling	Nasal sampling in airport and traveler questionnaire; airplane wastewater sampling; airport triturator;‡ air monitoring	Nasal sampling in airport and traveler questionnaire; airplane wastewater sampling; airport triturator; air monitoring
Median (range) participants per week§	535 (19–1395)	1,434 (1,334–1,746)	1,217 (325–3,490)	6,320 (1,689–9,321)	7,249 (4,366–12,628)
Median (range) traveler countries of origin per week§	1	6	43 (6–87)	123 (56–138)	143 (116–161)
Wastewater samples collected	0	0	89	417	783
Air samples collected	0	0	0	95	438
Laboratory methods used	RT-PCR, amplicon-based sequencing	RT-PCR, amplicon-based sequencing	RT-PCR, amplicon-based sequencing, target enrichment sequencing	RT-PCR, dRT-PCR, amplicon-based sequencing, target enrichment sequencing	RT-PCR, dRT-PCR, amplicon-based sequencing, target enrichment sequencing
Pathogen targets	SARS-CoV-2	SARS-CoV-2	SARS-CoV-2, influenza A and B pilot	SARS-CoV-2, influenza A and B, RSV testing of nasal samples, air, and wastewater; *Mycoplasma pneumoniae* testing of nasal samples in response to global outbreak reports; mpox testing of airplane and triturator‡ wastewater	Expanded multipathogen enrichment sequencing panel for up to 66 viruses deployed for wastewater samples

*ATL, Atlanta Hartsfield-Jackson International Airport, Atlanta, Georgia, USA; BOS, Logan Airport, Boston, Massachusetts, USA; CDC, Centers for Disease Control and Prevention; dRT-PCR, digital reverse transcription PCR; EWR, Newark Liberty International Airport, Newark, New Jersey, USA; FIFA, Fédération Internationale de Football Association; JFK T4 and T8, John F. Kennedy International Airport Terminal 4 and Terminal 8, Queens, New York, USA; IAD, Washington Dulles International Airport, Dulles, Virginia, USA; LAX, Los Angeles International Airport, Los Angeles, California, USA; MIA, Miami International Airport, Miami, Florida, USA; RSV, respiratory syncytial virus; RT- PCR, reverse transcription PCR; SEA, Seattle-Tacoma International Airport, Seattle, Washington, USA; SFO, San Francisco International Airport, San Francisco, California, USA. †SARS-CoV-2 variants characterized by specific spike mutations-F to L at position 456 and R to T at position 346-enhancing their transmissibility and immune evasion capabilities. ‡A consolidation point that captures wastewater samples from multiple flights and does not include airport terminal waste. §Nasal swab sampling.

To address skepticism about motives of private firms engaging in public health partnerships and the safeguards needed to secure public trust (*12*,*13*), it is essential to acknowledge concerns openly, emphasize shared goals, implement ethical oversight, prioritize long-term commitments, and highlight successful partnerships like those implemented during the COVID-19 pandemic (*14*). The World Health Organization’s Global Genomic Surveillance Strategy for Pathogens with Pandemic Potential 2022–2032 underscores the value of multisectoral partnerships for its successful implementation (*15*). Furthermore, a report by the National Academies of Science, Engineering, and Medicine on optimizing public–private partnerships for clinical cancer research highlights that successful partnerships should focus on the public good, address large-scale problems and unmet needs, leverage the strengths of each sector beyond what either could achieve on its own, and promote the generation of information, knowledge, or data for the public use (*16*). The Traveler-based Genomic Surveillance program’s public–private partnership exemplifies those criteria, demonstrating that multisectoral partnerships can be vital to public health before, during, and after crises.
